# Diffusion approximation-based simulation of stochastic ion channels: which method to use?

**DOI:** 10.3389/fncom.2014.00139

**Published:** 2014-11-03

**Authors:** Danilo Pezo, Daniel Soudry, Patricio Orio

**Affiliations:** ^1^Centro Interdisciplinario de Neurociencia de Valparaíso, Universidad de ValparaísoValparaíso, Chile; ^2^Department of Statistics and the Center for Theoretical Neuroscience at Columbia UniversityNew York, NY, USA; ^3^Instituto de Neurociencia, Facultad de Ciencias, Universidad de ValparaísoValparaíso, Chile

**Keywords:** channel noise, stochastic simulation, ion channel, Langevin, conductance based models

## Abstract

To study the effects of stochastic ion channel fluctuations on neural dynamics, several numerical implementation methods have been proposed. Gillespie's method for Markov Chains (MC) simulation is highly accurate, yet it becomes computationally intensive in the regime of a high number of channels. Many recent works aim to speed simulation time using the Langevin-based Diffusion Approximation (DA). Under this common theoretical approach, each implementation differs in how it handles various numerical difficulties—such as bounding of state variables to [0,1]. Here we review and test a set of the most recently published DA implementations (Goldwyn et al., 2011; Linaro et al., 2011; Dangerfield et al., 2012; Orio and Soudry, 2012; Schmandt and Galán, 2012; Güler, 2013; Huang et al., 2013a), comparing all of them in a set of numerical simulations that assess numerical accuracy and computational efficiency on three different models: (1) the original Hodgkin and Huxley model, (2) a model with faster sodium channels, and (3) a multi-compartmental model inspired in granular cells. We conclude that for a low number of channels (usually below 1000 per simulated compartment) one should use MC—which is the fastest and most accurate method. For a high number of channels, we recommend using the method by Orio and Soudry (2012), possibly combined with the method by Schmandt and Galán (2012) for increased speed and slightly reduced accuracy. Consequently, MC modeling may be the best method for detailed multicompartment neuron models—in which a model neuron with many thousands of channels is segmented into many compartments with a few hundred channels.

## Introduction

Understanding the effect of stochastic phenomena on the behavior of the nervous system requires stochastic simulation algorithms that effectively and accurately capture the dynamics of the underlying modeled phenomena. Among the sources for variability, the stochastic opening and closing of ion channels has caught the attention of several works over the past years. The best description of stochastic gating of ion channels is attained with the use of continuous time, discrete states Markov Chain (MC) processes (Neher and Stevens, 1977; Colquhoun and Hawkes, 1981), however this approach can be very slow in simulations with a large number of channels.

As an alternative to the explicit MC simulation, the Diffusion Approximation (DA) calculates the trajectory of a population of independent MCs using a Stochastic Differential Equation (SDE), sometimes called the Chemical Langevin Equation (Gillespie, 2000, 2007). Its application to the simulation of stochastic ion channels was suggested almost 20 years ago (Fox and Lu, 1994; Fox, 1997), but in the beginning there were some errors in the application of the scheme. This led to the belief that the approximation was not good enough (Mino et al., 2002; Bruce, 2007, 2009). Later, revised implementations of the algorithms were published showing that indeed the DA can reproduce the statistical properties of a population of discrete ion channel fluctuating between open and closed states (Goldwyn et al., 2011; Goldwyn and Shea-Brown, 2011). Other works (Dangerfield et al., 2012; Orio and Soudry, 2012) also offered a simplified description of the algorithm, making it easy to apply to any given kinetic scheme.

What the SDE system approach does is to approximate the trajectory in time of the fraction of channels at every state. For the simulation to remain physically meaningful, none of the fractions can be negative or greater than 1. We call this the “boundary constraint.” This constraint would occasionally break in the numerical simulation of the SDE, if we use its naively discretized form (the Euler–Maruyama method). This is because stochastic fluctuations can make the variables leave the [0,1] interval. The problem amplifies when the number of channels is low and stochastic fluctuations increase.

If the boundary constraint is not maintained, this can generate additional technical problems in the simulation. Specifically, the calculation of the stochastic terms involves the square root of a term involving the fraction variables, which may yield complex values when the variables are negative. Such complex values must be avoided. Therefore, we get another constraint, which we call the “Real-valued Square Root” (RSR) constraint. This constraint is automatically fulfilled if the boundary constraint is maintained.

Finally, the sum of the fractions over all states must be equal to 1 at all times. We call this the “normalization constraint.” Although this constraint is supposed to be guaranteed by the continuous-time equations (see below), machine rounding errors of the discretized equations can gradually break it. Additionally, any method that deals with the boundary constraint must also take normalization into account. For example, a naive truncation of any variable that leaves the [0,1] interval would break the normalization constraint.

To address these issues, a number of improvements have been proposed to the DA schemes.

Orio and Soudry (2012) proposed to allow the variables to freely change, ignoring the boundary constraint. In order to take care of the normalization constraint one of the variables was replaced with one minus the sum of the others. Additionally, an absolute value operation was added in the stochastic terms to maintain the RSR constraint. Previously, Goldwyn et al. (2011) also allowed the variables to freely change, but instead used a steady state approximation on the voltage to maintain the RSR constraint. However, such an approximation can be rather inaccurate (Dangerfield et al., 2012; Orio and Soudry, 2012; Huang et al., 2013a), even when the number of channels is relatively high.

Two methods aim to maintain the boundary constraint. Dangerfield et al. (2012) proposed that if variables break either the boundary or normalization constraint, they are “reflected” back into the valid region, in which all the constraints are kept. This is done using projection into a simplex (Chen and Ye, 2011). Huang et al. (2013a) proposed a different method. When the boundary constraint is broken, the variables should first be truncated. This breaks normalization, so the variables are then renormalized. Finally, in the next time step, the variables are incremented with the remainders from the truncation in the previous steps. As the boundary constraint is almost constantly broken, normalization is continuously corrected in both Dangerfield et al. (2012) and Huang et al. (2013a).

Other methods have been proposed with a different goal in mind. Schmandt and Galán (2012) aimed to reduce computational complexity and speeding up the simulation. They proposed to neglect stochastic noise terms in all state transitions, except those connecting the open state (or states), an approximation they call “Stochastic shielding.” Güler (2013) introduced a stochastic HH model with colored noise in the conductance terms as well as in the current terms in order to capture the non-trivial cross-correlation between the transmembrane voltage fluctuation and the component of open channel fluctuation attributed to multiple number of gates in individual ion channels. Another recently published method (Linaro et al., 2011), also used colored noise in the current terms (but not in the conductance terms). However, Linaro's method will not be examined here, because it involves a steady-state approximation in the stochastic terms (similarly to Goldwyn et al., 2011), which was shown to introduce inaccuracies (Orio and Soudry, 2012).

Despite the improvement in accuracy or computational efficiency that the new methods represent for the simulation of stochastic ion channel activity, we were concerned about the comparisons performed between them and the real benefit of implementing the numerical algorithms.

First, there is the computational cost issue. The initial motivation for developing DA methods was to make stochastic simulations faster than MC modeling. Therefore, if the extra computation needed to normalize and bound the variables makes it slower than MC then the purpose is defeated. Moreover, we already noticed that when the number of channels is low (when DA becomes more inaccurate) or with very small integration times, MC modeling can run faster than DA (see Figure 7 in Orio and Soudry, 2012). This, added to the fact that bounding and normalization of the DA requires more coding (and eventually, debugging), can render DA less attractive.

Second, we noted that the standard test employed to prove the accuracy of numerical methods for stochastic ion channels is the original Hodgkin and Huxley (HH) model. This was the only model used for testing in most previous papers, including a recent review (Rowat and Greenwood, 2014). This model, as standard and general as it is, reproduces the kinetics of ion channels of the squid axon at 6.3°C, thus differing greatly from mammalian nerve excitable membranes. This difference can be very significant, as we noted (Orio and Soudry, 2012). There we found that the use of a steady-state approximation in the stochastic terms usually does not introduce severe inaccuracies in the context of the original HH model. However, deviations were detected in common current clamp-based simulations when the steady-state approximation is used in a model inspired in mammalian (therefore faster) ion channels. It is noteworthy that the difference between mammalian inspired and the squid axon model relies only in the parameters that describe the transition rate constants (and thus the time scale of the model), while the equations and the model framework are identical.

Thus, we see a necessity for testing the DA with and without the recently proposed corrections in a wider spectrum of simulation scenarios and taking into account other variables than simulation accuracy, namely:

- To test the algorithms in models with faster kinetics than Hodgkin and Huxley (time scales of mammalian neurons) and models with geometry, where the number of ion channels in different compartments may differ.
- To quantify the real advantage of DA, and specifically its accuracy vs. its computational cost in comparison to MC.

In an attempt to test the real usability of the algorithms in the context of more complex neuronal models, we implemented them in one of the choice tools for biophysically-inspired modeling, the Neuron simulation environment (Hines and Carnevale, 2001; Carnevale and Hines, 2006). We conducted both single-compartment and multi-compartment simulations using MC or DA algorithms and compared their performance as well as the ability of different DA implementations to reproduce the variability introduced by MC modeling.

In our results all DA algorithms deviate to some degree from the MC modeling when the number of channels falls below 1000, regardless of the attempts to deal with normalization and bounding of the variables. However, we see that in this condition MC modeling runs, in most of the scenarios, faster than DA implementations. Therefore, one of the most common motivations to use DA, which is to achieve faster computation times, is not accomplished when the number of channels is low. However, when the number of channels is high, DA algorithms can accurately reproduce MC, with improved speed. Specifically, in this regime, no inaccuracy was detected in both Orio and Soudry (2012) and Huang et al. (2013a); Schmandt and Galán (2012) was slightly inaccurate; Güler (2013) was somewhat inaccurate; and Dangerfield et al. (2012) was the least accurate. In terms of computational speed, the ranking is as follows (see **Figure 8**): (1) Stochastic Shielding (Schmandt and Galán, 2012) (2) Colored Noise (Güler, 2013) (3) Unbound DA (Orio and Soudry, 2012) (4) Reflected DA (Dangerfield et al., 2012) (5) Truncated and Restored DA (Huang et al., 2013a).

## Materials and methods

### Simulations: models employed and tests performed

#### Original Hodgkin and Huxley model

The original Hodgkin and Huxley (HH) model (Hodgkin and Huxley, 1952) was simulated with the equation:

(1) $$\begin{array}{l} C_{m}\frac{dV(t)}{dt}=-g_{Na}(t)\left(V(t)-E_{Na}\right)-g_{K}(t)\left(V(t)-E_{K}\right)\\ \;-g_{l}\left(V(t)-E_{l}\right)+I_{stim}(t) \end{array}$$

With the exception of Güler's colored noise algorithm, sodium and potassium channels were treated as 8- and 5-state MCs, respectively. The corresponding kinetic schemes are:

$$\begin{array}{l} \begin{array}{ccccccc} m_{0}h_{0} & \overset{3\alpha_{m}}{\underset{\beta_{m}}{⇌}}\; & m_{1}h_{0} & \overset{2\alpha_{m}}{\underset{2\beta_{m}}{⇌}} & m_{2}h_{0}\; & \overset{\alpha_{m}}{\underset{3\beta_{m}}{⇌}} & m_{3}h_{0}\\ \beta_{h}↿⇂\alpha_{h} & \; & \beta_{h}↿⇂\alpha_{h} & \; & \beta_{h}↿⇂\alpha_{h} & \; & \beta_{h}↿⇂\alpha_{h}\\ m_{0}h_{0} & \overset{3\alpha_{m}}{\underset{\beta_{m}}{⇌}} & m_{1}h_{0} & \overset{2\alpha_{m}}{\underset{2\beta_{m}}{⇌}} & m_{2}h_{0} & \overset{\alpha_{m}}{\underset{3\beta_{m}}{⇌}} & m_{3}h_{0} \end{array}(\text{scheme1})\\ \;n_{0}\overset{4\alpha_{n}}{\underset{\beta_{n}}{⇌}}n_{1}\overset{3\alpha_{n}}{\underset{2\beta_{n}}{⇌}}n_{2}\overset{2\alpha_{n}}{\underset{3\beta_{n}}{⇌}}n_{3}\overset{\alpha_{n}}{\underset{4\beta_{n}}{⇌}}n_{4}\;(\text{scheme2}) \end{array}$$

Sodium and potassium conductances at time *t* (*g*_*Na*_(*t*) and *g*_*K*_(*t*)) were calculated as the fraction of channels in the conducting states *m*_3_*h*_1_ and *n*_4_ multiplied by the maximum conductances *g*_*Na*_ and *g*_*K*_, respectively. The kinetic rates α and β are given (in ms^−1^) by:

$$\begin{array}{l} \alpha_{m}(V)=\frac{0.1\left(V+40\right)}{1-\exp\left(-\frac{V+40}{10}\right)};\;\beta_{m}\left(V\right)=4\exp\left(-\frac{V+65}{18}\right)\\ \alpha_{h}(V)=0.07\exp\left(-\frac{V+65}{20}\right);\beta_{h}(V)=\frac{1}{1+\exp\left(-\frac{V+35}{10}\right)}\\ \alpha_{n}(V)=\frac{0.01\left(V+55\right)}{1-\exp\left(-\frac{\left(V+55\right)}{10}\right)};\;\beta_{n}(V)=0.125\exp\left(-\frac{V+65}{80}\right), \end{array}$$

where the terms were corrected to adjust the resting potential to −65 mV. Correspondingly, the rest of parameters are: *C*_*m*_ = 1μF/cm^2^, *g*_*Na*_ = 120 mS/cm^2^, *g*_*K*_ = 36 mS/cm^2^, *g*_*l*_ = 0.1 mS/cm^2^, *E*_*Na*_ = 50 mV, *E*_*K*_ = −77 mV, *E*_*l*_ = −54.3 mV.

With this model, the following tests were conducted:

- A 500-s simulation in the absence of any input. When the number of sodium channels is in the order of 20,000 (and lower), spontaneous firing starts to occur. We recorded the spike events and calculated the mean firing rate and the distribution of inter-spike intervals (ISIs).
- 15-s current clamp with 2-ms stimulus. The stimulus current was applied with 1 ms delay. Afterwards, 12 additional ms were simulated and the occurrence and timing of an action potential was recorded. The current amplitude varied from 0 to 15 μA/cm^2^ and 10,000 simulations were performed for each amplitude. Then, the firing efficiency, mean action potential time and variance of action potential time were calculated.
- Voltage clamp with action potential trace. A noisy voltage trajectory of 100 ms (including an action potential) was produced by simulating the HH model with the UA algorithm. Then, this trajectory was used as input to a stochastic model and the number of open channels in time was recorded. 2000 simulations were run and the mean and variance of open channels at each time was calculated. Additionally, the same procedure was performed with a deterministic HH model, thus allowing to obtain the expectation of open sodium and potassium channels, *E*[*Na*_*O*_](*t*) and *E*[*K*_*O*_](*t*). The expected variance was also calculated as *var* [*Na*_*O*_](*t*) = *E*[*Na*_*O*_](*t*) (1 − *E*[*Na*_*O*_](*t*))/*N*_*Na*_ and *var*[*K*_*O*_](*t*) = *E*[*K*_*O*_](*t*) (1 − *E*[*K*_*O*_](*t*))/*N*_*K*_. The results of the stochastic simulations were then compared to this exact solution.

#### Schmidt-Hieber and Bischofberger model—single compartment

The Schmidt-Hieber and Bischofberger (SB) model was proposed after the characterization of sodium channels both at the soma and at the axon initial segment of granule cells of the hippocampus (Schmidt-Hieber and Bischofberger, 2010). Sodium channels are described by the following kinetic scheme:

$$\begin{array}{ccccccc} m_{0}h_{0} & \overset{\alpha_{1}}{\underset{\beta_{1}}{⇌}} & m_{1}h_{0} & \overset{\alpha_{2}}{\underset{\beta_{2}}{⇌}} & m_{2}h_{0} & \overset{\alpha_{3}}{\underset{\beta_{3}}{⇌}} & m_{3}h_{0}\\ \beta_{h}↿⇂\alpha_{h} & & \beta_{h}↿⇂\alpha_{h} & & \beta_{h}↿⇂\alpha_{h} & & \beta_{h}↿⇂\alpha_{h}\\ m_{0}h_{0} & \overset{\alpha_{1}}{\underset{\beta_{1}}{⇌}} & m_{1}h_{0} & \overset{\alpha_{2}}{\underset{\beta_{2}}{⇌}} & m_{2}h_{0} & \overset{\alpha_{3}}{\underset{\beta_{3}}{⇌}} & m_{3}h_{0} \end{array}(\text{scheme3})$$

where the kinetic rates are given by

$$\begin{array}{l} \alpha_{i}(V)=\alpha_{i,0}\exp(\alpha_{i,1}V)\\ \beta_{i}(V)=\beta_{i,0}\exp(-\beta_{i,1}V)\\ \alpha_{h}(V)=\frac{\alpha_{h,0}}{1+\alpha_{\text{h},\text{1}}\exp(\alpha_{h,2}V)}\\ \beta_{h}(V)=\frac{\beta_{h,0}}{1+\beta_{h,1}\exp(\beta_{h,2}V)} \end{array}$$

Parameters α_*i*,*j*_ and β_*i*,*j*_ are given in Table 1 for both axonal and somatic channels. In single-compartment simulations, somatic parameters were employed.

**Table 1:** **Activation parameters for somatic and axonal sodium channels in the Schmidt-Hieber and Bischofberger model**.

**Parameter**	**Somatic channels**	**Axonal channels**	**Parameter**	**Somatic channels**	**Axonal channels**
α_1,0_(ms^−1^)	45.850	62.648	β_1,0_(ms^−1^)	0.0144	0.00194
α_1,1_(mV^−1^)	0.00239	0.0116	β_1,1_(mV^−1^)	0.0885	0.1377
α_2,0_(ms^−1^)	19.808	34.783	β_2,0_(ms^−1^)	0.5650	0.0957
α_2,1_(mV^−1^)	0.02218	0.0299	β_2,1_(mV^−1^)	0.06108	0.0928
α_3,0_(ms^−1^)	71.812	76.698	β_3,0_(ms^−1^)	0.7531	1.2488
α_3,1_(mV^−1^)	0.0659	0.0537	β_3,1_(mV^−1^)	0.0365	0.0311
α_*h*,0_(ms^−1^)	0.5757	6.882	β_*h*,0_(ms^−1^)	2.8301	3.573
α_*h*,1_	162.84)	4654.0	β_*h*,1_	0.289	01933
α_*h*,2_(mV^−1^)	0.0268	0.0296	β_*h*,2_(mV^−1^)	0.0696	0.07496

Potassium channels in SB model are simulated by the same kinetic scheme as HH model (scheme 2) with the following voltage dependent transition rates:

$$\begin{array}{l} \alpha_{n}(V)=sc\times 0.01\frac{V+55}{1-\exp\left(-\frac{V+55}{10}\right)}\\ \beta_{n}(V)=sc\times 0.125\exp\left(-\frac{V+65}{80}\right) \end{array}$$

where *sc* is a scale parameter that adjusts the kinetic constants according to the compartments in which the channels are being simulated. For single compartment simulations, equation (1) was used with the following parameters: *C*_*m*_ = 1μF/cm^2^, *g*_*Na*_ = 20 mS/cm^2^, *g*_*K*_ = 4 mS/cm^2^, *g*_*l*_ = 0.1 mS/cm^2^, *E*_*Na*_ = 75 mV, *E*_*K*_ = −95 mV, *E*_*l*_ = −70 mV. With this model, the number of channels was controlled by the membrane area, given a unitary conductance of 20 pS/cm^2^. We tested areas ranging from 15.7 to 628 μm^2^, resulting in 157 to 6283 sodium channels and 31 to 1257 potassium channels.

With this model, the following tests were conducted at different values of membrane area:

- 20-s Iclamp with 1-ms stimulus: the stimulus current was applied with 1 ms delay. Afterwards, an additional 18 ms were simulated and the occurrence and timing of an action potential was recorded. The current amplitude varied from 0 to 8 μA/cm^2^ and 10,000 simulations were performed for each amplitude. Then, the firing efficiency, mean action potential time and variance of action potential time were calculated.
- Voltage clamp with action potential trace: the same procedure described for the HH model.

#### Schmidt-Hieber and Bischofberger model—idealized multicompartment model

An idealized model similar to the one described in Schmidt-Hieber and Bischofberger (2010, see **Figure 5A**) was simulated using stochastic algorithms for the ion channels. The parameters for ion channel densities and kinetic constants were used as described in the article with minor modifications, such as the absence of an axonal bleb and a longer axon for some simulations.

### Simulation algorithms

The methods described and tested here are designed to simulate a number of independent and identical Markov Chains (MCs) with a discrete number of states, keeping track of the number of channels in any state at any given time. For the description of the algorithms, we denote S as the total number of states in a MC, *i* ∈ {1, …, *S*} are the individual states, and N_i_ is the number of MCs in state i.

#### Markov chain simulations (MC)

Markov chains were simulated using the Stochastic Simulation Algorithm (SSA) (Gillespie, 1976) with some modifications. Briefly, the method consists in:

At time *t*, calculate the effective transition rate λ(*t*) as

$$\lambda (t)=\sum_{i}^{S}N_{i}(t)\zeta_{i}(t)$$

where ζ_*i*_(*t*) is the sum of transition rates for transitions escaping from state *i*.

Calculate the time for the next transition *t*_*n*_ as

$$t_{n}=t_{p}-\frac{\log\xi_{1}}{\lambda (t)}$$

where *t*_*p*_ is the time of the previous transition (0 at the beginning of the simulation) and ξ_1_ is a random number uniformly distributed within [0,1], drawn after the previous transition.

If *t*_*n*_ > *t*, continue integrating the time and the membrane voltage equation.

If *t*_*n*_ < *t*, perform a transition:

Calculate the probability of all *j* transitions:

$$P_{j}(t)=\frac{N_{i}(t)\alpha_{j}(t)}{\sum_{j}N_{i}(t)\alpha_{j}(t)}$$

where *i* is the state originating transition *j* and α_*j*_ its rate.

Build a cumulative sum of all transition probabilities. Draw a random number ξ_2_ uniformly distributed in [0,1] and find the first term in the cumulative probability that is greater than ξ_2_.

Execute the transition indicated by the term found in the previous step, and draw a new random number ξ_1_ to be used for the time of the next transition.

#### Unbounded diffusion approximation (UA) (Orio and Soudry, 2012)

The DA algorithm was implemented with SDEs described previously (Orio and Soudry, 2012; see also Mélykúti et al., 2010). In matrix form, the equations for the sodium and potassium channels are, respectively:

$$\frac{dX_{Na}}{dt}=A_{Na}X_{Na}+\frac{1}{\sqrt{N_{Na}}}S_{Na}(X_{Na})\xi {\left(t\right)}_{Na}$$

(2) $$\frac{dX_{K}}{dt}=A_{K}X_{K}+\frac{1}{\sqrt{N_{K}}}S_{K}(X_{K})\xi {\left(t\right)}_{K}$$

where *X*_*Na*_ = [*m*_0_*h*_0_ *m*_1_*h*_0_ *m*_2_*h*_0_ *m*_3_*h*_0_ *m*_0_*h*_1_ *m*_1_*h*_1_ *m*_2_*h*_1_ *m*_3_*h*_1_]^*T*^ and *X*_*K*_ = [*n*_0_ *n*_1_ *n*_2_ *n*_3_ *n*_4_]^*T*^ are column vectors with the fraction of channels at any given state, and ξ(*t*)_*Na*_ and ξ(*t*)_*K*_ are column vectors of independent normally distributed random variables (mean 0, variance 1) with length 10 and 4, respectively. *N*_*Na*_ and *N*_*K*_ are the number of sodium and potassium channels, respectively. The rate matrices *A*_*Na*_ and *A*_*K*_ and square root matrices *S*_*Na*_(*X*_*Na*_) and *S*_*K*_(*X*_*K*_) can be directly found from the state diagram of the corresponding ion channel type. This is explained in detail around equations 1 and 13 and the Supplemental Material in Orio and Soudry (2012). For example, in the case of Potassium channels we have

$$A_{K}=\left[\begin{array}{ccccc} -4\alpha_{n} & \beta_{n} & 0 & 0 & 0\\ 4\alpha_{n} & -3\alpha_{n}-\beta_{n} & 2\beta_{n} & 0 & 0\\ 0 & 3\alpha_{n} & -2\alpha_{n}-2\beta_{n} & 3\beta_{n} & 0\\ 0 & 0 & 2\alpha_{n} & -\alpha_{n}-3\beta_{n} & 4\beta_{n}\\ 0 & 0 & 0 & \alpha_{n} & -4\beta_{n} \end{array}\right]$$

and

$$\begin{array}{l} S_{K}\left(X_{K}\right)=[\begin{array}{cc} \sqrt{4\alpha_{n}n_{0}+\beta_{n}n_{1}} & 0\\ -\sqrt{4\alpha_{n}n_{0}+\beta_{n}n_{1}} & \sqrt{3\alpha_{n}n_{1}+2\beta_{n}n_{2}}\\ 0 & -\sqrt{3\alpha_{n}n_{1}+2\beta_{n}n_{2}}\\ 0 & 0\\ 0 & 0 \end{array}\;\;\;\;\;\;\;\\ \;\;\;\;\;\;\begin{array}{cc} 0 & 0\\ 0 & 0\\ \sqrt{2\alpha_{n}n_{2}+3\beta_{n}n_{3}} & 0\\ -\sqrt{2\alpha_{n}n_{2}+3\beta_{n}n_{3}} & \sqrt{\alpha_{n}n_{3}+4\beta_{n}n_{4}}\\ 0 & -\sqrt{\alpha_{n}n_{3}+4\beta_{n}n_{4}} \end{array}]. \end{array}$$

Note that in the case of the Schmidt-Hieber and Bischofberger model, the only difference from the HH model is that the rate constants for the sodium channel equations are different.

To take care of normalization, variables *m*_1_*h*_0_ … *m*_3_*h*_1_ and *n*_1_ … *n*_4_ were advanced by an Euler-Maruyama scheme and the remaining two were calculated as *m*_0_*h*_0_ = 1 − *m*_1_*h*_0_ − *m*_2_*h*_0_ − *m*_3_*h*_0_ − *m*_0_*h*_1_ − *m*_1_*h*_1_ − *m*_2_*h*_1_ − *m*_3_*h*_1_ and *n*_0_ = 1 − *n*_1_ − *n*_2_ − *n*_3_ − *n*_4_. As we do not control the bounding of the variables between 0 and 1, in order to ensure real-valued square roots we calculated the stochastic terms *S*(*X*) (and only those terms) taking the absolute value of the variables. Thus, we refer to this algorithm as UA – Unbounded with Absolute values in stochastic terms.

#### Reflected SDEs (Ref) (Dangerfield et al., 2012)

This method aims to normalize the variables *m*_1_*h*_0_ … *m*_3_*h*_1_ and *n*_1_ … *n*_4_ and to keep them bounded in the interval [0, 1] using the reflected stochastic equation approach, described in section V of Dangerfield et al. (2012). We have used this method together with the DA equation system (Equations 2).

#### Truncated and restored DA (TR) (Huang et al., 2013a)

We used the DA equations (Equations 2) with an additional residual term. This residual term was introduced to ensure the boundary and normalization constraints, as explained in section II.F in Huang et al. (2013a).

#### Stochastic shielding approximation (SSmc) (Schmandt and Galan, 2012)

In this method, transitions not connecting to the conducting states are approximated to be deterministic and solved as ODEs. Transitions connecting conducting with non-conducting states are solved as Markov Chains with the already mentioned algorithm. As there is a mixture of continuous (ODEs) and discrete (MC) treatment of variables, violations of the constraints occur. In our implementation, and inspired by the code by Schmandt and Galan, variables going off the [0,N] boundary are manually corrected and normalization was performed as in the UA algorithm.

#### Stochastic shielding approximation with DA (SSda)

We modified the Schmandt and Galán (2012) approach by calculating the stochastic transitions with a DA approach (Equations 2) rather than using MCs. Therefore, SSda similarly uses the same DA equations, but the stochastic terms related to transitions not connecting to the conducting states were neglected.

For example, for potassium channels, we now used

$$S_{K}(X_{K})=\left[\begin{array}{cccc} 0 & 0 & 0 & 0\\ 0 & 0 & 0 & 0\\ 0 & 0 & 0 & 0\\ 0 & 0 & 0 & \sqrt{\alpha_{n}n_{3}+4\beta_{n}n_{4}}\\ 0 & 0 & 0 & -\sqrt{\alpha_{n}n_{3}+4\beta_{n}n_{4}} \end{array}\right],$$

Thus, we needed only 2 Brownian terms for sodium channels and 1 for potassium channels. Boundary and normalization constraints were again treated as in the UA algorithm, that is: there was no bounding of the variables; the absolute value of the variables was used in the square roots of the stochastic terms; and normalization was applied by calculating *m*_0_*h*_0_ = 1 − *m*_1_*h*_0_ − *m*_2_*h*_0_ − *m*_3_*h*_0_ − *m*_0_*h*_1_ − *m*_1_*h*_1_ − *m*_2_*h*_1_ − *m*_3_*h*_1_ and *n*_0_ = 1 − *n*_1_ − *n*_2_ − *n*_3_ − *n*_4_.

#### HH with colored noise terms (CN) (Güler, 2013)

We simulated the Güler's Brownian harmonic oscillator using the system of stochastic differential equations 6.1, 6.2 in Güler (2013), using the constant parameters given in Table 2 in Güler (2013). Note that these equations are very different from the standard DA equations.

To take care of the normalization constraint at each time step, if any of the variables *m*, *h* or *n*, left the [0,1] interval, then the stochastic term η was redrawn until the variable fulfilled the boundary constraint.

### Software

All the models and simulations algorithms presented here were implemented and run in the Neuron simulation environment (Hines and Carnevale, 2001; Carnevale and Hines, 2006). The different algorithms were written inside the MOD files for each channel. With the exception of TR, all algorithms run fine regardless of the numeric integrator specified (cnexp or euler). They also produce the same results in Python using the Euler-Maruyama integration method (tested in some selected cases). The TR algorithm required the specification of the Euler integrator within the MOD file to produce the results presented here, otherwise a much lower firing rate was obtained. Simulation control and the recording of variables were specified with Python scripts (Hines et al., 2009). Sample codes and.mod files can be found in ModelDB (http://senselab.med.yale.edu/ModelDB/ Accession 167772).

Data analysis and plotting was performed using the Python libraries numpy, scipy, and matplotlib.

## Results

To test the accuracy of the methods we performed a series of simulations, comparing the variability of the results to that obtained with explicit MCs solved by the exact Gillespie algorithm (Gillespie, 1976, 2007). We employed the original Hodgkin and Huxley model (Hodgkin and Huxley, 1952), in order to reproduce previously published comparisons of the algorithms. In addition we performed some tests with a faster model, based on sodium channels from granular cells in the hippocampus (Schmidt-Hieber and Bischofberger, 2010). Finally, we simulated a model neuron with multiple compartments (Schmidt-Hieber and Bischofberger, 2010) and measured the variability in the generation *and conduction* of action potentials.

### Simulations with the original Hodgkin and Huxley model

#### Firing variability—15 ms simulation with stimulus

**Figure 1:**
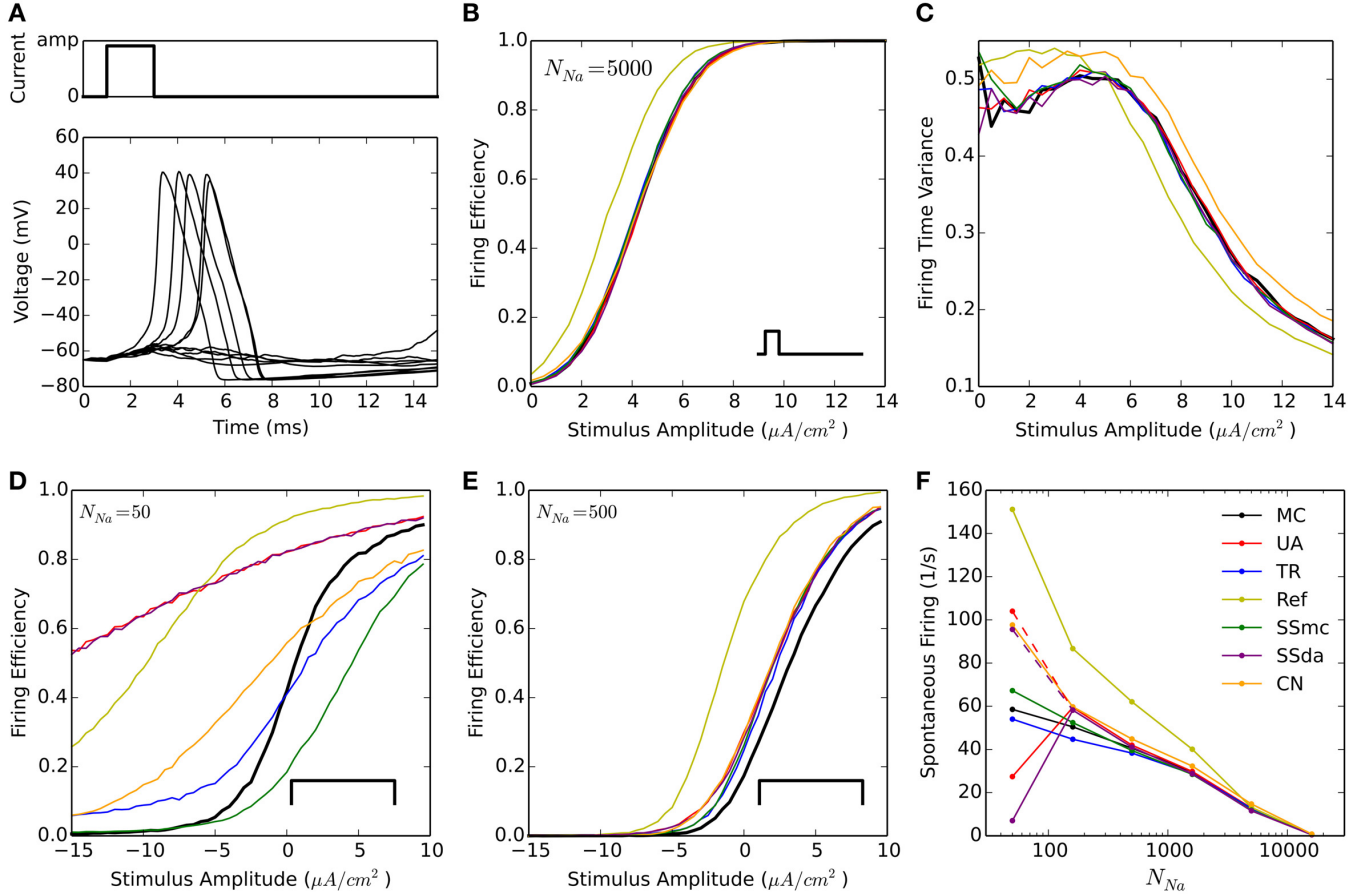
**Stochastic simulations of the HH model in current clamp configuration. (A)** 10 sample voltage traces obtained with the DA simulation, showing the stimulus at the top. Amp = 4.5 μA/cm^2^. **(B)** Firing efficiency, expressed as the fraction of simulations in which an action potential was elicited, out of 10,000 sweeps. Simulations performed with *N*_*Na*_ = 5000, *N*_*K*_ = 1500. The inset represents the type of stimulus. **(C)** Variance of the firing time at different amplitudes of the pulse, for the same simulations shown in **(B)**. **(D,E)** Firing efficiencies obtained with a constant pulse (inset) of the indicated amplitudes with *N*_*Na*_ = 50, *N*_*K*_ = 15 **(D)** and *N*_*Na*_ = 500, *N*_*K*_ = 150 **(E)**. **(F)** Mean number of spikes per seconds obtained in 500 s simulations (*dt* = 0.5 μs) without stimulus at different number of sodium channels *N*_*Na*_. *N*_*K*_ = 0.3 ∗ *N*_*Na*_. Segmented lines represent data obtained with *dt* = 0.1 μs (UA) and *dt* = 0.02 μs (SSda) The algorithms are indicated as follow: MC, Markov chains (Gillespie's algorithm); UA, unbounded DA with absolute values in the stochastic terms (Orio and Soudry, 2012); TR, truncated and restored DA (Huang et al., 2013a); Ref, reflected DA (Dangerfield et al., 2012); SSmc, stochastic shielding approximation (Schmandt and Galán, 2012); SSda, stochastic shielding with DA approximation; CN, colored noise (Güler, 2013).

A widely used test to compare stochastic simulation algorithms (Mino et al., 2002; Bruce, 2007; Orio and Soudry, 2012 and others) consists of a short simulation (15 ms) in which a 2-ms current stimulus is given after a 1-ms delay (Figure 1A). Depending on the amplitude of the stimulus the probability of eliciting an action potential increases, and this relationship depends on the number of channels. Figure 1B shows the probability of firing an action potential in 10,000 trials at different stimulus amplitudes, for the algorithms tested with *N*_*Na*_ = 5000. Figure 1C plots the variance of action potential timing, a measure of jitter. The Reflection method produces a higher firing probability at all stimulus amplitudes. This entails a lower variability in action potential timing. Additionally, Güler's CN method produces a higher variability than MC and other methods. This result is repeated with higher number of channels, however at a lower number of channels the spontaneous firing of action potentials makes the comparison unreliable. To compare the behavior of the models with *N*_*Na*_ ≤ 1600, we modified the protocol so the stimulus is sustained during the simulation (Figure 1D, inset) and explored negative values of current amplitude. As can be seen in Figures 1D,E, the behavior of all the DA algorithms (as well as of SSmc) deviates from MC considerably for *N*_*Na*_ = 50 and to a minor degree for *N*_*Na*_ = 500. Again, the Reflection method produces a higher firing probability than the other methods.

#### Spontaneous firing rate

The original HH model with stochastic ion channel produces spontaneous firing activity that increases as the number of channels is decreased. With each simulation algorithm and with sodium channel number (*N*_*Na*_) ranging from 50 to 50,000, we simulated 500 s and recorded the occurrence of action potentials. Figure 1F shows the mean frequency of spikes that were detected in the simulations. With *N*_*Na*_ ≥ 1600, almost all the methods reproduce the behavior of MC modeling. The sole exception is the Reflection method, that showed a higher firing probability at *N*_*Na*_ = 1600 and below. To discard some incompatibility of the Neuron simulation environment with the reflection procedure, we repeated this simulation using an Euler-Maruyama integration procedure written in Python and obtained the same result. Huang's truncated and restored DA method seems to be the one that more closely follows MC modeling at extremely low number of channels, only slightly underestimating the firing rate. At *N*_*Na*_ = 160, the Unbound DA and Stochastic Shielding methods overestimate the firing rate, dropping abruptly when *N*_*Na*_ = 50. This latter behavior is actually due to numeric overflows that made the simulations run without producing action potentials. This was corrected using a smaller time step (Figure 1F, segmented lines). Thus, the Unbound DA (note that SSda is also unbound) becomes numerically unstable when the number of channels is too low.

#### Voltage clamp—noisy voltage trace with action potential

**Figure 2:**
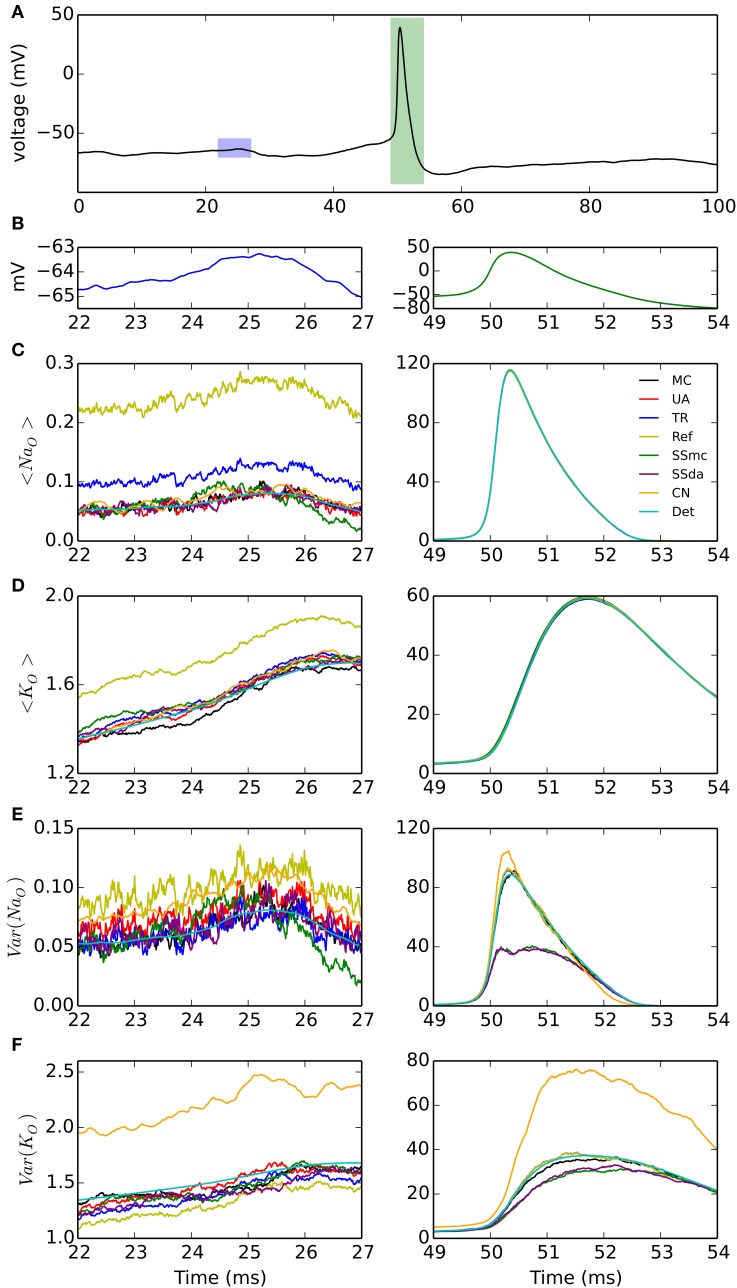
**Simulations of the stochastic HH model under voltage clamp. (A)** Voltage trace applied to simulated channels in a 100-ms simulation, repeated 2000 times. Blue and green rectangles represent the 5-ms intervals that are expanded in the left and right columns of the figure, respectively. **(B)** Detail of the voltage traces corresponding to the time windows analyzed in **(C–F)**. Note the different vertical scales. **(C)** Mean of open sodium channels during the subthreshold (left) and action potential (right) regimes, for the simulation algorithms tested. **(D)** Mean of open potassium channels. **(E)** Variance of open sodium channels. **(F)** Variance of open potassium channels. *N*_*Na*_ = 500, *N*_*K*_ = 160. Very similar results were obtained with *N*_*Na*_ = 5000, *N*_*K*_ = 1600 (see text).

A third test, to check how the different DA methods can reproduce the variability of channel openings obtained with MC modeling, consists on recording the response of the model channels to a fixed voltage trajectory obtained from a stochastic simulation. The voltage trace is shown in Figure 2A and it contains an action potential as well as a noisy background (zoomed in Figure 2B). With each model and condition, 2000 independent simulations were run and the time evolution of open channels was recorded. At each point in time, the mean and variance of the open channels was calculated. In addition to the comparison with the behavior of MC simulation, we compared to the expected mean of open channels which is calculated by applying the same voltage clamp simulation to deterministic HH channels. Moreover, we can compute the expected variance as explained in Methods.

The results are shown in Figure 2. During the subthreshold regime, Reflection method overestimates the mean of open channels, both for sodium and potassium (Figures 2C,D, left). Huang's TR algorithm also overestimates the mean of open sodium channels (Figure 2C, left) to a minor extent. However, during the action potential any difference between the DA methods and the MC modeling or the exact solution appears to be negligible (Figures 2C,D, right). Regarding the variance of the open channels (Figures 2E,F), the main deviation seems to occur with Güler's CN algorithm, which overestimates the variance of both open sodium and open potassium channels, during the subthreshold regime (left) and the action potential (right). Schmandt's stochastic shielding approximation (both SSmc and SSda) underestimates the variance of open channels during the action potential, when the voltage changes more rapidly. The results shown here are for *N*_*Na*_ = 500, *N*_*K*_ = 160; with higher number of channels (*N*_*Na*_ = 5000, *N*_*K*_ = 1600) we naturally observed less fluctuations but the results maintained: Reflected DA overestimates the mean of open channels in subthreshold regime; Colored Noise overestimates the variance of open channels in subthreshold regime; and Stochastic Shielding underestimates the variance of open channels during the action potential (not shown).

### Schmidt-Hieber and Bischofberger model—single compartment

We decided to use a model with faster sodium channels, resembling mammalian ion channels, to test the accuracy of the DA methods when the transitions between states occur at faster rates. We chose a recently published model that focuses on the fast opening of sodium channel in the axon initial segment of granule cells from the hippocampus (Schmidt-Hieber and Bischofberger, 2010). We will refer to this model as the “SB” model. We noted that the SB model does not show spontaneous firing when simulated stochastically. Therefore, the 500-ms simulation test was not performed.

#### Firing variability—20 ms with 1 ms stimulus

**Figure 3:**
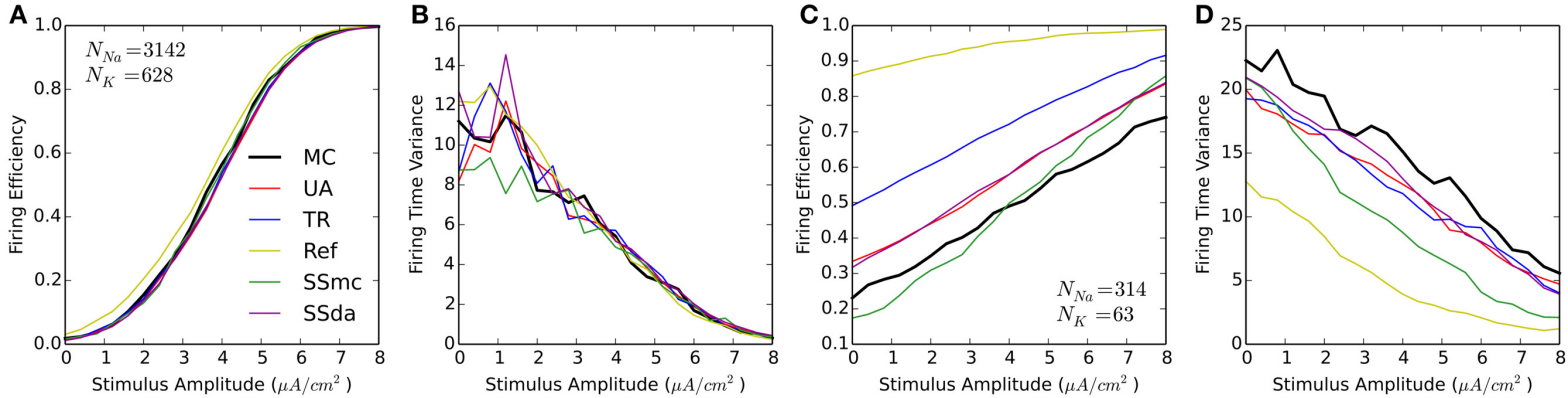
**Responses of the Schmidt-Hieber and Bischofberger (SB) single compartment model to a 1-ms stimulus pulse. (A,B)** Firing efficiency **(A)** and Variance of the firing time **(B)** for the stochastic model with a membrane area of 314.2 μm^2^ and the number of channels indicated. 2000 sweeps were simulated and the Firing Efficiency is the fraction of sweeps in which an action potential was elicited. **(C,D)** Same as **(A,B)** with a membrane area of 31.4 μm^2^.

We performed the test in which 20-ms were simulated with a 1-ms current stimulus (similar to Figure 1A). For each stimulus amplitude, 2000 simulations were run and the Firing Efficiency, mean firing time, and firing time variance were calculated. Figure 3 shows that the results are similar to that obtained with HH. At high number of channels, most methods perform reasonably similar to MC with a higher excitability of the Reflection method (Figures 3A,B). However, when the number of channels is low all DA methods fail to approximate the results of MC, showing a much higher probability of firing at all amplitudes of the stimulus (Figure 3C). Also, the Reflection method shows a lower firing time variability (Figure 3D).

#### Voltage clamp

We applied the voltage clamp test with the same voltage trace as the HH model to the SB model stochastic channels. Similar to what we observed for HH model, the Reflection method overestimates the mean of open channels (both sodium and potassium) during the subthreshold regime. Huang's truncated and restored method also overestimates it to a minor degree. During the action potential, the greatest deviation occurs with the stochastic shielding approximation, which underestimates the variance for both channels. With a higher number of channels, we observed similar results, with the exception of Huang's TR method performing better in the mean of open channels (not shown).

### Schmidt-Hieber and Bischofberger model—multi-compartment simulations

**Figure 4:**
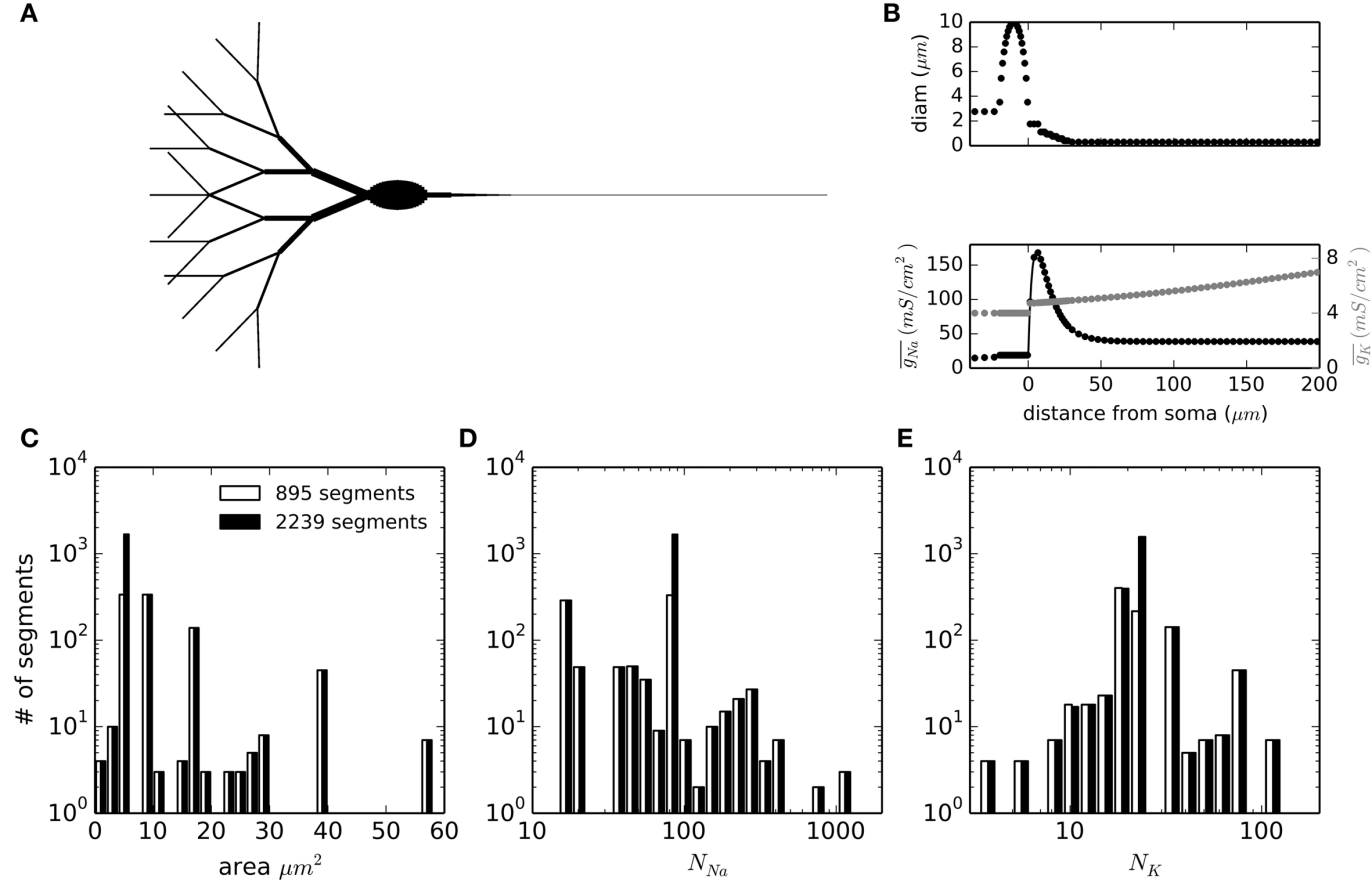
**Geometry and compartment statistics of the multicompartment model. (A)** Schematic representation of the neuron simulated. The image is not to scale, and longer sections have been shortened for illustration purposes (specially the axon). Total soma length is 20 μm and its widest diameter is 10 μm. Dendrite tips are 300 μm from the soma and the total axon length is 1500 μm (895 segments) or 7500 μm (2239 segments). **(B)** Diameter of sections and density of sodium and potassium conductance as a function of the distance from the beginning of the axon (negative distances correspond to the soma and dendrites). **(C–E)** Distribution histograms for the membrane areas **(C)**, the number of sodium channels **(D)** and the number of potassium channels **(E)** along the segments (compartments) in which the model is discretized.

To test the applicability of DA methods in more complex simulations of physiological relevance, we set up a multi-compartmental model of a neuron. We chose the idealized neuron described in Schmidt-Hieber and Bischofberger (2010) and shown in Figure 4A. Moreover, we kept the particular inhomogeneous sodium channel density for the axon that causes the action potentials to be initiated in the axon initial segment (AIS), about 10 μm from the soma (Figure 4B, bottom). The neuronal sections were spatially discretized according to their spatial constant λ, with a further increase in the number of segments in the AIS area. In total, the number of segments simulated were 895 with a 1500 μm axon and 2239 when the axon was extended to 7500 μm. The distributions of segment areas is shown in Figure 4C. Together with the different ion channel densities and a unit conductance of 20 pS, the resulting distributions of number of channels per segment are shown in Figures 4D,E. It is noteworthy that the number of channels to be simulated in any given segment is rarely higher than 500 for sodium channels and never higher than 120 for potassium channels. In the model with the long axon, this adds up to 171189 sodium channels and 50670 potassium channels.

**Figure 5:**
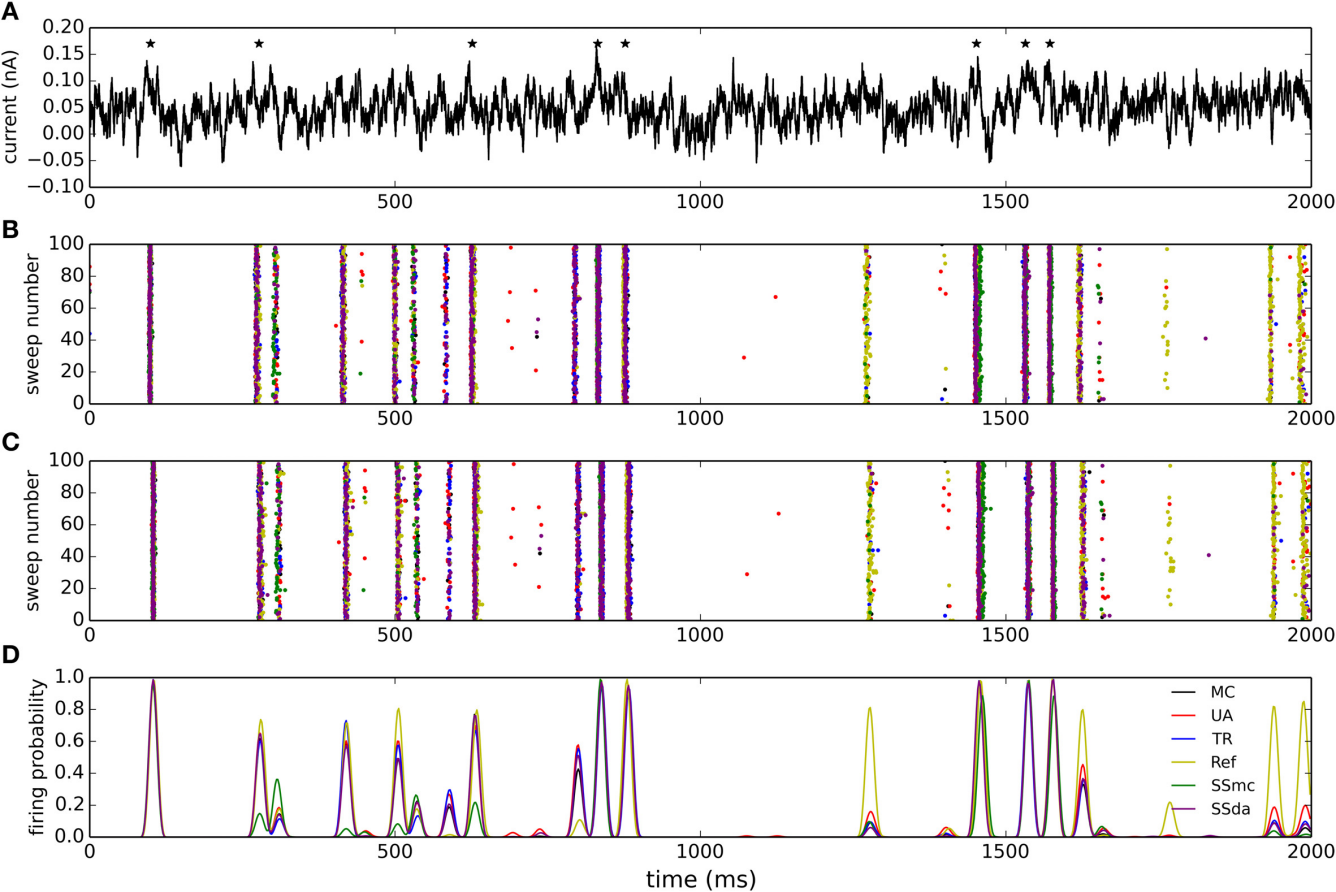
**Stimulation of the multi-compartmental model with a noisy current injection. (A)** Noisy current employed to stimulate the model. The trace was obtained as an Örnstein-Uhlenbeck process with τ = 5 ms. The mean of the depicted trace is 0.05 nA and the standard deviation is 0.03 nA. Stars denote the times at which action potentials are elicited in a deterministic simulation.**(B)** Raster plots of action potentials detected at the soma during 100 of the simulations performed with each algorithm (from a total of 400). The color of the dots represents the algorithms according to the legend in **(D)**. **(C)** Raster plots of action potentials detected at the tip of the axon. **(D)** Smoothed normalized firing probability obtained from the 400 simulations. The rasters were discretized in bins of 2 ms, adding 0.0025 for each action potential detected in a bin. The resulting vectors were then smoothed by convolving with a Blackmann filter function of length 20.

**Figure 6:**
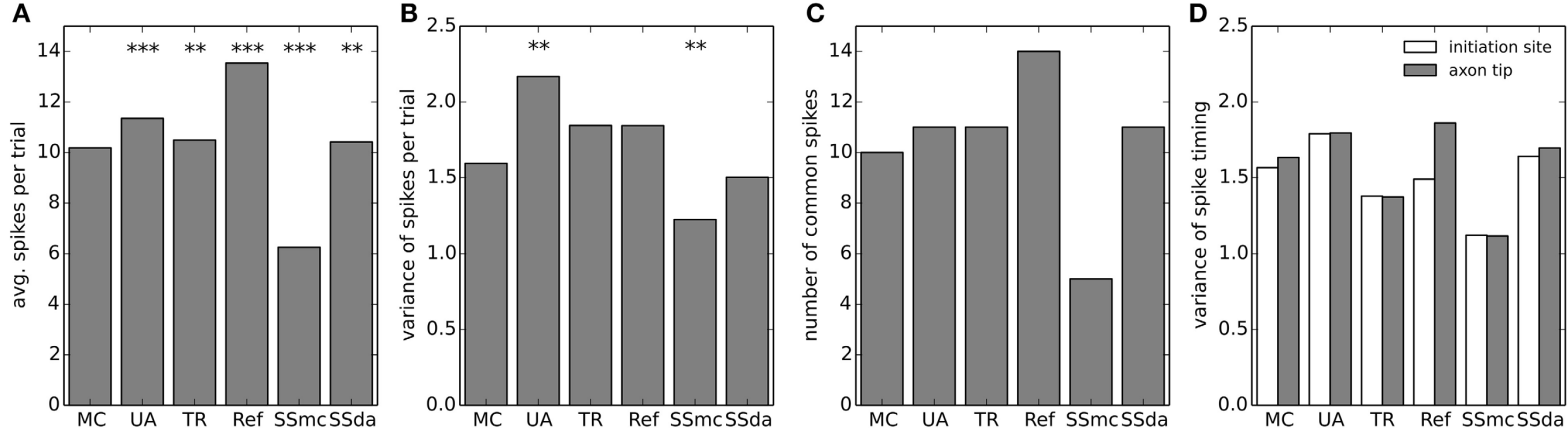
**Firing statistics and variability in the multi-compartment model. (A)** Mean of spikes per sweep detected at the axon tip in each of the stochastic simulation algorithms. Data was compared to MC using a *t*-test. **(B)** Variance of the spikes per trial in each stochastic simulation. Data was compared to MC using a Bartlett's test for equal variance. **(C)** Number of common spikes found in each simulation. A common spike is a spike present in more than 50% of the trials with the same timing, considering a window of ±5 ms. **(D)** For all the common spikes detected, the variance in their timing was calculated near the initiation site (blue bars) and at the axon tip (green bars). In **(A–B)**, ^***^p < 0.001, ^**^p < 0.01.

The model neuron with different stochastic channels was subject to a current clamp stimulus applied to the soma. The stimulus consisted in a 2 s noisy stimulus (Figure 5A) which in a deterministic simulation elicited 8 action potentials (represented by stars in the Figure). 400 independent simulations were performed with the same stimulus and Figures 5B,C show the raster plots (100 simulations) of spikes detected at the soma and at the tip of the axon, respectively. Figure 5D depicts a normalized firing probability calculated for the spikes at the tip of the axon. Both raster and firing probability plots show that the simulations with the Reflection method displayed a greater excitability, as several action potentials were only elicited with this algorithm and were not seen with the other DA methods or were seen with a much lower probability (i.e., around *t* = 1300 ms, *t* = 1700 ms and near the end of the trace). On the other hand, Stochastic Shielding with MC produced a lower excitability, firing near half of the action potentials per sweep than the other methods. The mean of spikes per sweep (Figure 6A) was significantly different to MC for all the algorithms, not only the Reflection and SSmc methods. With the Reflection method, however, the deviations from the other methods go beyond a higher excitability. Some spikes fired with high probability with all DA methods except for Reflection (see for instance around 800 ms), and some spikes had a slightly different timing with Reflection (700, 1500 ms). Therefore, the Reflection method in this test actually introduced a bias, producing spikes with different timings than the other DA method. The variability of the number of spikes elicited per trial varied with some DA algorithms compared to MC (Figure 6B) but only in the case of UA and SSmc a significant difference was observed.

We took the raster plots and searched for spikes that were repeated in at least 50% of the sweeps with the same timing ± 5 ms. These were called “common spikes” and for most algorithms 10–11 common spikes were found (Figure 6C), with the exception of Reflection and SSmc methods. Then, we measured how variable the timing of these spikes was at two axon locations, one near the initiation site and the other at the tip (Figure 6D). Although the TR method reproduced more closely the variability obtained with MCs, none of the observed differences was significant.

**Figure 7:**
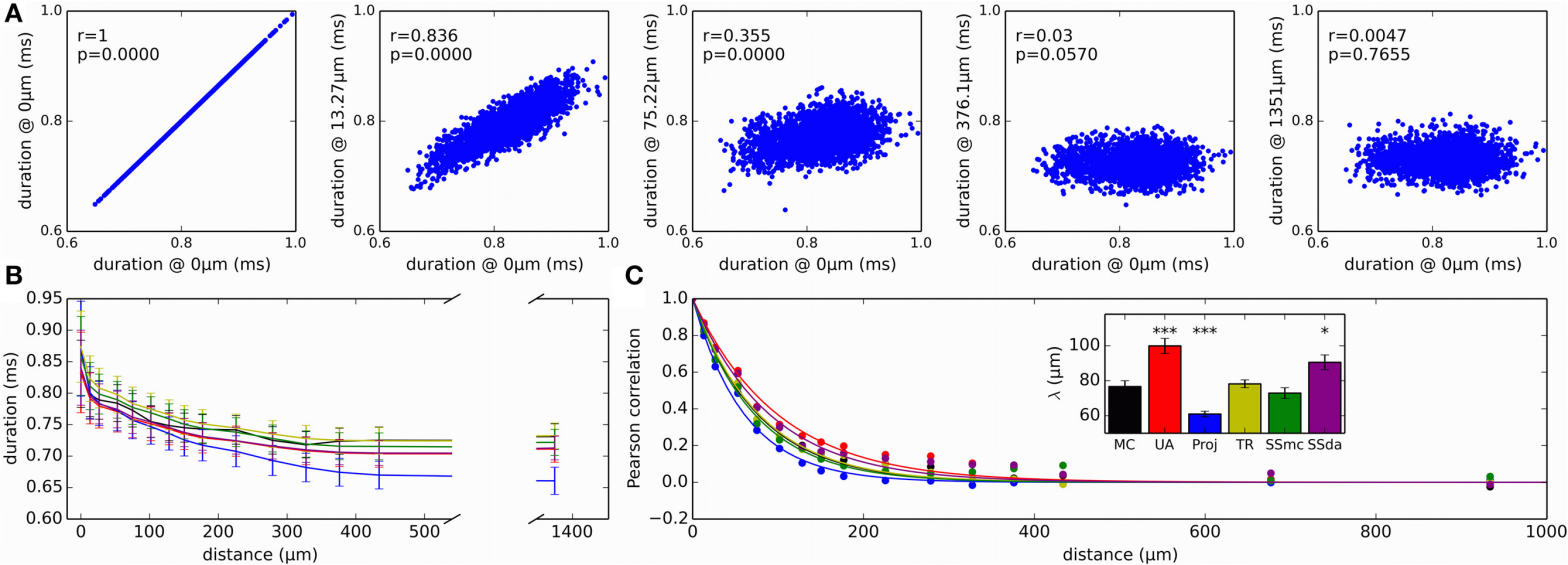
**Variability and correlations in the duration of action potentials. (A)** Duration of all action potentials (measured at 0 mV) at different positions of the axon, plotted against the duration at a site near the initiation site. This reference location is designated as “0 μm.” Data presented correspond to simulation with MC. *r* is the Pearson correlation coefficient and p is the associated *p*-value when testing for a correlation different to 0. **(B)** Evolution of action potential duration along the axon, for the different simulation algorithms. Data is mean ± *SD*. **(C)** Correlation of action potential duration at different points in the axon, with the duration at the reference “0 μm” location. Lines represent the fit to a single exponential decay. Inset: length constants λ obtained in the fit of the different data sets. Error bars represent the *SD* of the parameter estimation. The fits were compared to MC using an extra sum of squares *F* test, to test the hypothesis that each data set and the MC set could be fit with the same parameter λ. ^***^p < 0.001, ^*^p < 0.05.

As a measure of variability with functional consequences, we examined how the duration of the action potential (duration measured at the detection threshold level of 0 mV) evolved as it propagates along the axon. The relevance of this measure is that the duration of action potentials at the release zone of a synapse will impact the amount of neurotransmitter released. Figure 7A plots the duration of all the action potentials recorded in a MC simulation at several sites of the axon, plotted against the duration at a site near the initiation. As a first observation, in the reference site there is a wide distribution of action potential durations, which gets narrower as the measurement site moves along the axon. Also, action potentials are shorter in the distal axon than in the initiation site and the Reflection method produces the shorter action potentials of all the methods (Figure 7B).

Besides getting shorter, action potential duration at the distal axon is completely uncorrelated to the duration at the initiation site (Figure 7A, right). We looked at how the correlation of action potential duration decays along the axon with the different stochastic simulation algorithms. Results are shown in Figure 7C. It is apparent that Reflection method produces a faster decay in the correlation, while the Unbound DA produces a longer propagation of correlation. To test for similarity, we fitted an exponential decay to the data points, obtaining a space constant λ. An extra sum of squares *F* test was performed to test the null hypothesis that the data points of each set could be fitted with the same λ as the MC data, showing that Unbound DA, Reflection and SSda methods produced a behavior significantly different to that obtained with MC (Figure 7C, inset). When the test was repeated with a fit to a double exponential decay, the same result was obtained.

### Computation time

**Figure 8:**
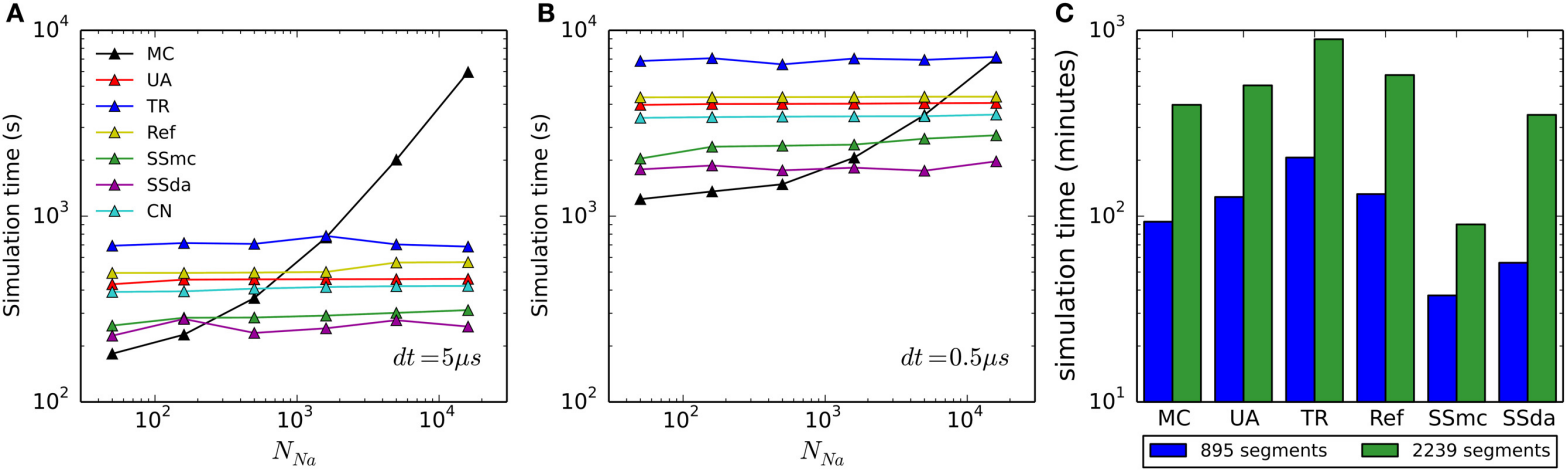
**Computational cost of the simulation algorithms. (A,B)** Real time needed to perform a 500-s current clamp simulation of the HH model with each algorithm and different numbers of channels, using an integration time step (*dt*) of 5 μs **(A)** or 0.5 μs **(B)**. **(C)** Real time needed to perform a 2-s simulation of the full multi-compartment model, with an axon of length 1500 μm (blue) or 7500 μm (green). All simulations (single- and multi-compartment) were run in a 2.5 GHz AMD Opteron 6378 processor. Only one core was used for each simulation.

To account for the usefulness of the simulation algorithms, we found important to compare the computational cost of each of them. Figures 8A,B show the time required to simulate 500 ms of the HH model with an integration time step (*dt*) of 5 and 0.5 μs, respectively, for each algorithm used. As we reported previously (Orio and Soudry, 2012), DA methods are highly sensitive to *dt* but mostly insensitive to the number of channels. On the other hand, MC simulations are sensitive to both, but its sensitivity to the number of channels approaches a linear relationship as the number of channel increases. Importantly, as both *dt* and/or number of channel decrease, MC outperforms all of the DA methods, giving the best simulation times precisely in the condition where the DA methods showed to be more problematic.

In the case of the multi-compartment simulation, MC is faster than all DA methods except for Schmandt's stochastic shielding approximation (Figure 8C). This result is not surprising, given that the number of channels per simulated compartment was almost always below 1000 (Figures 6C,D).

## Discussion

In this work we numerically tested five Diffusion Approximation algorithms proposed to reproduce the behavior of a number of simultaneous Markov Chains, in the context of stochastic ion channel modeling. Most of these algorithms (Dangerfield et al., 2012; Orio and Soudry, 2012; Schmandt and Galán, 2012; Huang et al., 2013a) are based on a Langevin Equation proposed for stochastic modeling of the Hodgkin and Huxley model (Fox and Lu, 1994; Fox, 1997). However, they deal in different ways with numerical issues that appear in the simulation of stochastic trajectories: the requirement that the variables be bounded in [0,1] (“boundary constraint”) and the requirement that at any given time the sum of variables must be equal to 1 (“normalization constraint”). The boundary constraint breaks more frequently when the number of channels being simulated is low or when the integration time step increases, because the stochastic terms scale with $\sqrt{dt/N}$. Therefore, special attention should be put to the performance of these simulation algorithms—and their capacity of reproducing MC behavior—with a low number of channels.

Our tests were primarily aimed with a practical question in mind: what method should be used to study the contribution of channel stochasticity to neural excitability in any given specific context? To answer this question, the simple test of the numerical accuracy is not enough. Modeling algorithms should also be examined for applicability and simplicity of implementation in different contexts and for the computational efficiency for the intended model to be solved.

### Accuracy

Regarding accuracy, in brief we found that all DA algorithms fail in the reproduction of MC behavior when the number of channels is low (generally speaking, below 1000), with no clear “winner.” For example, the TR procedure improved the results in some current clamp simulations (Figures 1D–F), but introduced higher deviations than the UA in others (Figure 3). The Reflection method in our simulations performed the worse, introducing a higher firing probability in current clamp simulations. Most likely, this is related to the higher mean of open sodium channels observed in voltage clamp (Figure 2C).

When the number of channels is 5000 or higher, the TR, UA and SS methods perform well in reproducing MC behavior in current clamp tests. This means that in the high number of channels regime, bounding the variables to [0,1] is not essential, as the UA and SSda implementations (both unbounded) give the same results than TR. Inaccuracies were observed (Figure 1) in the Reflection method, and, to a lesser extent, the CN method. Additionally, both methods showed inaccuracy in the voltage clamp tests. Also on these tests, the SS methods showed some inaccuracy in the variance of open channels during the action potential, but the results in current clamp simulations suggest that this may not be relevant for the neural excitability.

The results with the multicompartment model deserve special attention, because some results were different to what was observed in a single compartment. Although the SSmc method introduced only minor inaccuracies in the single compartment test, this method severely altered the excitability of the multicompartment model. We could not identify the reason for this effect, and tested several alternatives of bounding and normalization which did not improve the results. Nevertheless, the overall effect is a reduced excitability, which is consistent with the deviation observed in single compartment (Figure 1D). We confirmed that this effect does not arise from our implementation of the algorithm, by repeating the single compartment test with the Matlab code published by Schmandt and Galán (ModelDB acc. 144468) and obtaining similar results (not shown). So, although it is an attractive method for increasing simulation speed in multicompartmental models, its use is not recommended until further testing is performed. The Reflection method altered the excitability of the neuron but the results were in agreement with the single compartment results. Curiously, the use of Stochastic Shielding in a DA framework (SSda) improves the behavior of the simulations, bringing it closer to the behavior of MCs, like the TR does.

### Applicability and simplicity

In terms of applicability, a first observation is that the colored noise approach (Güler, 2013) can be used only if the ion channel is composed of independent subunits. This is sometimes true (e.g., the original HH model), but not always (e.g., the SB model). For example, channels with non-identical voltage sensors (Vandenberg and Bezanilla, 1991; Horn et al., 2000), cooperativity in the movement of voltage sensors (Bezanilla et al., 1994; Schoppa and Sigworth, 1998) or complex allosteric gating mechanisms (Horrigan and Aldrich, 2002) do not have independent subunits. Therefore, they cannot be modeled with the colored noise approach. Besides, Güler's equations have a number of constant parameters (γ_*K*_, γ_*Na*_, ω^2^_*K*_, ω^2^_*Na*_, *T*_*K*_, *T*_*Na*_) that were estimated empirically to obtain an adequate level of channel noise (Güler, 2013). It is not clear how these parameters can be derived for other ion channels, even if they are composed of identical and independent gating subunits.

The other DA-based algorithms can be applied to any given kinetic scheme but first require to obtain the corresponding system of SDEs. Until recently, it seemed quite complicated to implement, since the original descriptions involved the calculation of a matrix root square (Fox and Lu, 1994; Goldwyn et al., 2011). However, alternative derivations of the Langevin equation (Mélykúti et al., 2010; Orio and Soudry, 2012) yield an explicit form that does not use complex matrix operations. This method can be derived for any given kinetic scheme using simple and intuitive rules without using matrix notation (see Supplemental Material in Orio and Soudry, 2012). Importantly, any kinetic scheme can be translated to an SDE system and the equations can be written explicitly. This makes it simpler to employ low-level or limited languages such as C or Neuron's NMODL, which are compiled prior to execution code and therefore run faster.

The DA algorithms differ in the treatment of boundary and normalization constraints. In this regard, the Unbound DA (UA) is the simplest, not taking care of the boundaries issue and doing a simple normalization by making one variable to depend on the others (alternative normalization procedures can be implemented, for instance dividing all the variable values by their sum). Finally, to avoid non-real square roots in the stochastic terms, a simple absolute value operation is performed. The Stochastic Shielding approximation, when used with DA equations (SSda), can simplify the code even further because it uses less stochastic terms. Huang's truncation and restoration is also rather simple to follow and implement. However, it requires several lines of code and a series of nested *if* and *for* blocks when written in simple languages. As a side note, we found that the restoration procedure requires the specification of the Euler integration method within Neuron's NMODL files. Failing to do so, using instead the default Crank-Nicholson integrator, results in severely distorted results such as a much lower firing rate. Dangerfield's reflection method takes similarly amount of lines of code as TR but it was more complicated to follow and implement.

### Computational efficiency

Our comparison of simulation time shows that DA-based methods are not the best choice for all the situations. It has already been noted that when the number of channels is low, MC modeling runs faster than DA (Orio and Soudry, 2012). The limit (number of channels) at which this happens is variable, depending not only on the time step used for numerical integration (as shown in Figure 8A vs. Figure 8B) but also on the kinetics of the channels being simulated, as this determines the number of transitions occurring in MCs (see below). Interestingly, the Stochastic Shielding in the context of MC (SSmc) behaves like the DA methods: it is faster than MC only with large numbers of channels and its performance depends mainly on the integration time step. This results from the increase in the number of (non-stochastic) ODEs that have to be solved at each integration step, regardless of the actual transitions that occur.

At first glance, it seems that a similar comparison carried out in another work (see Figure 8 in Huang et al., 2013b) produced very different results. In Huang et al. the MC method is faster than DA only if there are less than 20 ion channels. In contrast, we found that MC is more efficient if there are less than ~500 channels (*dt* = 5 μs) or ~5000 channels (*dt* = 0.5 μs). The main reason for this large difference is that in the MC simulations of Huang et al. the state dynamics of each gating particle were updated individually. This method is highly inefficient and should be avoided in stochastic simulations of neurons. In contrast, we used the standard efficient MC method by Gillespie (1976), which tracks only the total number of channels in each state.

Interestingly, in another work (Rowat and Greenwood, 2014) it was found that Güler's CN method is much faster than Unbound DA. However, we have found that it is only a little faster (Figures 8A,B). The difference might be attributed to the language (Neuron vs. Python), or some other difference in the implementation.

Within all DA methods, Stochastic Shielding is the fastest in all circumstances. Next comes the colored noise approach (as noted, not applicable to all kinetic schemes) closely followed by Unbounded DA and Reflection. Recall that Stochastic Shielding (SSda) produces a minor loss in accuracy when used with a large number of channels, being in almost all cases indistinguishable from MC modeling. Finally, in our simulations the less efficient (slowest) algorithm was the Truncated and Restored DA (Huang's).

The numerical stability issue also deserves to be considered. Although we did not perform a systematical assessment of numerical stability in our simulations, we noted that simulations with the UA and SSda algorithms produced unreliable results with *N*_*Na*_ = 50, even at the lowest *dt* of 0.5 μs. As the other algorithms did not show this problem, it is most likely due to the lack of variable bounding. We did not pursue in finding a fix for these fails as the low channel number is a condition where MC modeling becomes the fastest and most accurate method.

### Theoretical estimates of accuracy and speed

For a given model, when is it better to use DA instead of MC? Specifically, we would like to know in advance when a DA approach will be accurate, and also faster than MC. A definite answer usually requires some preliminary simulations. However, as we explain next, a rough estimate could be obtained based on the following numbers: the simulation timestep, the number of channels to be simulated, and their typical constant rates.

Suppose we have N ion channels (of some particular type), with X of these ion channels in some state A, and α being a kinetic rate from state A to another B. In each simulation timestep dt, let Δ be the number of channels switching from state A to state B. As different channels are independent, ∆ is distributed according to a binomial distriubtion with *n* = *X*, and *p* = α*dt*. Therefore, the average number of channels switching from A to B in that timestep is *np* = *X*α*dt*.

These quantities can be used to estimate the expected accuracy of DA. As explained in Orio and Soudry (2012), the key idea in DA is to use the central limit theorem and approximate the distribution of ∆ to be Gaussian. This approximation becomes accurate when *np* = *X*α*dt* ≫ 1. This also means that *N*α*dt* ≫ 1 since *X* < *N*. For example, in the HH model the slowest kinetic rates (in the relevant voltage range), are about α ~ 0.1*ms*^−1^. Then if *dt* = 5 μs DA should be expected to be accurate only when *N* > 2000, which is comparable to what we found in our simulations.

Using the same quantities we can also estimate the relative speed of the MC and DA algorithms. Simulation time is roughly proportional to the number of times the simulation variables (the fraction of channels in each) are being updated at each *dt* timestep (in which the voltage is updated). On the one hand, the MC algorithm performs updates each time a single channel switches between states. The number of these updates in each timestep, for each type of switch, is proportional to Δ. Recall that the mean of Δ is equal to *np* = *X*α*dt*. Therefore, in total, about *N*α*dt* updates are performed on each timestep (where α is the appropriate average over all the kinetic rates). On the other hand, the DA algorithms perform a single update at each timestep. Therefore, DA should become more efficient than MC only when *N*α*dt* > 1. For example, in the HH model most state transitions occur near rest voltage in the (fast) *m* kinetics, and so we get approximately α ~ 2ms^−1^. This condition yields results comparable to what we found in our simulations (Figure 8): if *dt* = 5 μs then N > 100, and if *dt* = 0.5 μs then N > 1000.

## Conclusions

Suppose *dt* is the simulation timestep, *N* is the number of ion channels, and α is the “typical” transition rate of the channel. Our results suggest that, as a rule of a thumb,

- If *N*α*dt* < 1, then MC simulation should be used—since it is both the fastest and most accurate method. Note that this is usually relevant to neuron models with less than 500 channels in a compartment—which is the common case in large multi-compartmental neuron models.
- If *N*α*dt* > 1, DA should be used. In this case, one should use the method by Orio and Soudry (2012) which allows the simulated variables to remain unbounded (with an absolute value used to keep the stochastic terms real-valued). Additionally, the stochastic shield method by Schmandt and Galán (2012) method can be used with the DA equations to further speed up simulation, while remaining reasonably accurate.

### Conflict of interest statement

The authors declare that the research was conducted in the absence of any commercial or financial relationships that could be construed as a potential conflict of interest.
